# Altered Synaptic Properties During Integration of Adult-Born Hippocampal Neurons Following a Seizure Insult

**DOI:** 10.1371/journal.pone.0035557

**Published:** 2012-04-23

**Authors:** Johanna Jackson, Deepti Chugh, Per Nilsson, James Wood, Karl Carlström, Olle Lindvall, Christine T. Ekdahl

**Affiliations:** 1 Laboratory of Neurogenesis and Cell Therapy, Wallenberg Neuroscience Center, Lund, Sweden; 2 Lund Stem Cell Center, Lund University, Lund, Sweden; 3 Inflammation and Stem Cell Therapy Group, Division of Clinical Neurophysiology, Lund, Sweden; Louisiana State University Health Sciences Center, United States of America

## Abstract

Pathological conditions affect several stages of neurogenesis in the adult brain, including proliferation, survival, cell fate, migration, and functional integration. Here we explored how a pathological environment modulates the heterogeneous afferent synaptic input that shapes the functional properties of newly formed neurons. We analyzed the expression of adhesion molecules and other synaptic proteins on adult-born hippocampal neurons formed after electrically-induced partial status epilepticus (pSE). New cells were labeled with a GFP-retroviral vector one week after pSE. One and three weeks thereafter, synaptic proteins were present on dendritic spines and shafts, but without differences between pSE and control group. In contrast, at six weeks, we found fewer dendritic spines and decreased expression of the scaffolding protein PSD-95 on spines, without changes in expression of the adhesion molecules N-cadherin or neuroligin-1, primarily located at excitatory synapses. Moreover, we detected an increased expression of the inhibitory scaffolding protein gephyrin in newborn but not mature neurons after SE. However, this increase was not accompanied by a difference in GABA expression, and there was even a region-specific decrease in the adhesion molecule neuroligin-2 expression, both in newborn and mature neurons. Neuroligin-2 clusters co-localized with presynaptic cholecystokinin terminals, which were also reduced. The expression of neuroligin-4 and glycine receptor was unchanged. Increased postsynaptic clustering of gephyrin, without an accompanying increase in GABAergic input or neuroligin-2 and -4 expression, the latter important for clustering of GABA_A_ and glycine receptors, respectively, could imply an increased but altered inhibitory connectivity specific for newborn neurons. The changes were transient and expression of both gephyrin and NL-2 was normalized 3 months post-SE. Our findings indicate that seizure-induced brain pathology alters the sub-cellular expression of synaptic adhesion molecules and scaffolding proteins related to particularly inhibitory but also excitatory synapses, which may yield functional consequences for the integration of adult-born neurons.

## Introduction

New hippocampal neurons are continuously generated in the adult human brain [1], where they may contribute to memory formation [2], [3]. In animal models, adult neurogenesis can be affected by various pathologies, including seizures [4], [5], [6], and it is detectable both in healthy and resected epileptic human brain tissue [1], [7].

In the intact brain, integration of new hippocampal neurons occurs over several months [8], with an initial tonic GABA-induced depolarization converting into GABA-mediated hyperpolarization at 3 weeks [9], and full maturity of excitatory synapses in the molecular layer (ML) at about 2 months . Simultaneously with the formation of dendrites on the new cells, their axons grow and establish functional glutamatergic synapses with hilar interneurons, mossy cells and CA3 pyramidal neurons [10].

The effect of epileptic conditions on hippocampal neurogenesis depends on seizure severity and magnitude of the associated inflammatory response [8] . After severe generalized status epilepticus (SE), new aberrant hilar neurons are frequent and may contribute to network hyperexcitability [6], [11]. Also, new cells located in the granule cell layer (GCL) show abnormal dendrites and spine density [12], [13], and may have an accelerated early excitatory functional integration [14]. In contrast, in less severe, partial SE (pSE) [15], the majority of new neurons remain in the GCL and few cells show abnormal dendrites [5]. Electrophysiological recordings reveal less excitatory and more inhibitory synaptic drive onto the new neurons born after pSE, compared to new cells which develop in response to running, suggesting that new neurons in the GCL may act to mitigate the abnormal seizure-induced hyperexcitability of the hippocampus [5].

When new neurons integrate into an existing neuronal network, their function is influenced by numerous and heterogeneous afferent synaptic inputs. In order to explore how a pathological environment may modulate these inputs, we decided to characterize the expression of adhesion molecules and other synaptic proteins at afferent synapses on the new neurons. The balance between excitation and inhibition, postulated as a mechanism underlying epileptogenesis [16], can be modified by synaptic adhesion molecules [17]. Adhesion molecules are important both during synaptic assembly and development and in synaptic signalling and plasticity [18], [19], [20], [21], [22]. Adhesion molecules also regulate the integration of adult-born neurons [23], [24] and N-cadherin, an adhesion molecule essential for target recognition and excitatory synapse stabilization [25], [26], [27], is increased in the hippocampus after generalized SE [28], [29]. However, apart from these initial studies, the synaptic expression of adhesion molecules on adult-born neurons *in vivo* and how it is modulated by pathological conditions is virtually unknown.

Here we have used confocal imaging of retrovirally-labeled cells combined with immunohistochemistry to determine cluster density and size of several adhesion molecules and other synaptic proteins on adult-born hippocampal neurons of different maturation stages in rats exposed to pSE and in non-stimulated controls, and in comparison to presumably mature neurons. We find prominent time-specific sub-cellular changes at both excitatory and inhibitory synapses, which may be important for the functional connectivity of the newly formed neurons in the pathological environment.

## Materials and Methods

### Ethics Statement

All experimental procedures followed guidelines set by the Malmö-Lund Ethical Committee for the use of laboratory animals and were conducted in accordance with European Union directive on the subject of animal rights.

### Animals

Adult male Sprague-Dawley rats (body weight 200–250 g, n = 97 Charles-River, Germany), were housed under 12 h light/dark cycle with food and water *ad libitum*.

### Induction of status epilepticus

**Figure 1:**
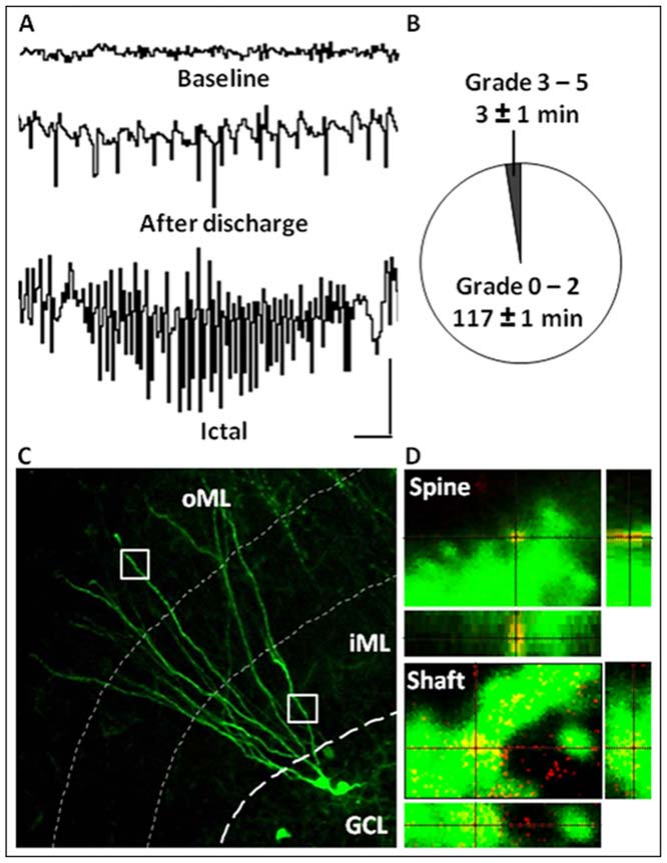
Inclusion criteria and methodology. *A*, EEG recordings from electrode-implanted animals showing baseline activity (top) before stimulations in the hippocampus, high-frequency ictal activity immediately following a stimulation period (afterdischarge) during the one hour of induction of status epilepticus (SE) (middle), and high-frequency ictal activity during the two hours post-stimulation period of self-sustained SE (bottom). *B*, Pie chart showing the time spent in partial (grade 0–2) and generalized seizures (grade 3–5) according to the Racine scale. *C*, Photomicrograph showing a representative GFP+ dendritic tree of an adult-born neuron extending into the molecular layer (ML) of the dentate gyrus. Cluster analyses on the GFP+ dendrites were carried out in two regions of interest of the dendritic tree, the inner and outer ML (iML and oML, respectively, marked by squares) to reflect different synaptic inputs onto the new cells. *D*, Orthogonal reconstructions from confocal z-series, viewed in *x-z* (bottom) and *y-z* (right) planes, were used to confirm co-localization of clusters on GFP+ dendrites, as shown here with a PSD-95 cluster on a dendritic spine (top) and a gephyrin cluster on the dendritic shaft (bottom). Calibration is 2 s and 1 mV (*A*). Scale bars are 20 µm (*C*) and 1 µm (*D*).

Animals were anesthetized with isofluorane (2%) and implanted unilaterally with a bipolar insulated stainless steel electrode (Plastics One, Roanoke, VA) into the right hippocampal ventral CA1–CA3 region for stimulation and recording (coordinates: 4.8 mm caudal and 5.2 mm lateral to bregma; 6.3 mm ventral from dura, toothbar set at −3.3 mm). Another electrode was positioned between the skull and adjacent muscle to serve as a reference. At seven days after electrode implantation, 48 rats were subjected to electrically-induced pSE [15], [30]. Forty-eight electrode-implanted but non-stimulated animals served as controls. Afterdischarge threshold was assessed by delivering a 1 s train of constant current balanced, biphasic, square-wave pulses (1 ms duration, 50 Hz) at an initial current of 10 µA (base-to-peak), and increased by 10 µA increments at 1 min intervals until at least a 5 s duration of afterdischarge was evoked. After 30 min, a 1 h suprathreshold stimulation with 10 s trains of 1 ms, 50 Hz, biphasic, square-wave pulses was applied. Stimulation was interrupted every 9th min for 1 min of electroencephalographic (EEG) recordings (Figure 1A). Only rats that exhibited self-sustained ictal EEG activity (i.e., SE) for 2 h after the cessation of stimulation were used in the study (Figure 1A). Behavioral convulsions were observed throughout the stimulation period and the following 2 h, and were rated using the classical 5-grade motor seizure scale [31]. Virtually all animals exhibited partial SE with less than 5% having generalized SE [15], as previously reported [5] (Figure 1B). Ictal activity was stopped with pentobarbital (65 mg/kg i.p.).

A less severe seizure model was used for comparisons of gephyrin and NL-2 expression on newly formed neurons at 6 weeks of age [32]. This model is characterised by mild seizures and minor accompanying inflammation [32].

### Labeling of new neurons

Seven days after induction of SE, rats were anesthetized with isofluorane, and injected with a retroviral vector expressing the GFP gene under the control of the CAG promoter (1.0–1.1 transducing units/ml) [33]. Two 1.5 µl-retroviral injections were made in the dorsal hippocampus contralateral to the electrode (coordinates: 3.6 mm caudal and 2.0 mm lateral to bregma, and 2.8 mm ventral from dura; 4.4 mm caudal and 3.0 mm lateral to bregma, and 3.0 mm ventral from dura; toothbar at −3.3 mm). Animals were either transcardially perfused or decapitated at one (n = 13), three (n = 33), or six weeks (n = 38), or three months (n = 13) after retroviral injections.

### Immunohistochemistry

Scaffolding proteins require weak fixation for optimal detection and their analysis is often limited by epitope-masking after tissue fixation, therefore for analysis of gephyrin and PSD-95 distribution, a specific fixation protocol was used (similar to Schneider et al. [34]). Rats were lightly anesthetized with isofluorane and decapitated. The hippocampus contralateral to the electrode was removed and placed in ice-cold, oxygenated artificial cerebrospinal fluid (aCSF) (95% O_2_/5% CO_2_, 295–300 mOsm, containing (in mM): 119 NaCl, 2.5 KCl, 1.3 MgSO_4_, 2.5 CaCl_2_, 26.2 NaHCO_3_, 1.0 NaH_2_PO_4_, and 11.0 glucose, pH = 7.2–7.4), cut in 300 µm coronal sections on a vibratome, and placed in gassed 33°C aCSF for 20 min and then in 4% paraformaldehyde (PFA) in 0.15 M phosphate-buffered saline (PBS) for 10 min. Sections were cryoprotected in 20% sucrose in 0.1 M PBS overnight, cut in 12 µm coronal sections on a cryostat, mounted onto gelatin-coated glass slides, and stored at −20°C for at least 1 h.

For analysis of morphology of GFP+ cells, adhesion molecules, presynaptic proteins, GABA, and postsynaptic glycine receptor, rats were given an overdose of pentobarbital (250 mg/kg i.p.) and transcardially perfused with 100 ml ice-cold saline and 200 ml ice-cold PFA (4% in 0.1 M PBS, pH = 7.4). Brains were removed, post-fixed overnight, dehydrated in 20% sucrose in 0.1 M PBS overnight, and then cut into 30 µm-thick coronal sections and stored in cryoprotective solution at −20°C.

For immunohistochemistry, the following primary antibodies were used: rabbit anti-GFP (1∶10000, Abcam, UK), mouse anti-gephyrin (1∶1000 (or 1∶500 for perfused tissue), Synaptic Systems, Germany), mouse anti-PSD-95 (1∶1000, Abcam), mouse anti-N-cadherin (1∶200, Abcam), goat anti-neuroligin-1 (NL-1) (Ab #1: 1∶100, Santa Cruz Biotechnology, Germany, Ab#2: 1∶1000, Synaptic Systems), goat anti-neuroligin-2 (NL-2) (1∶100, Santa Cruz Biotechnology), rabbit anti-neuroligin-4 (NL-4) (1∶500, Abcam), mouse anti-synaptophysin (1∶400, Sigma, Sweden), rabbit-anti-GABA (1∶2000, Sigma), mouse anti-VGLUT1 (1∶100, Synaptic Systems), rabbit anti-cholecystokinin (CCK) (1∶2000, Sigma), mouse anti-GlyR (1∶100, Synaptic Systems). Free-floating or mounted sections were incubated with the appropriate primary antibody overnight at +4°C and secondary antibody for 2 h at room temperature. Staining for the adhesion molecules, presynaptic vesicular markers, GABA, and glycine receptor involved an antigen retrieval step of incubating the sections in sodium citrate buffer at 90°C for 20 min. Secondary antibodies were: Cy3-conjugated donkey anti-mouse/rabbit/goat (1∶200, Jackson ImmunoResearch, UK), FITC-conjugated goat anti-rabbit (1∶200, Jackson ImmunoResearch) or biotinylated goat anti-chicken (1∶200, Vector laboratories, UK), and Alexa-488 conjugated streptavidin (1∶200, Jackson ImmunoResearch). Free-floating sections from perfused animals were mounted on gelatin-coated microscope slides and coverslipped with DABCO (Sigma).

### Morphological analysis

Cell counting and morphological analysis were performed ipsilaterally to the virus injections in 4–6 hippocampal sections by an observer blind to the treatment conditions as previously described [35] using an Olympus BX61 epifluorescence microscope. GFP+ cells were counted in the subgranular zone (SGZ)/GCL and dentate hilus for analysis of relative distribution of cell bodies within these areas. For all GFP+ cells, axon exit point, dendrite exit points, and total number of dendrites leaving the cell soma were analyzed. Dendritic polarity was determined by classifying the angles of the dendrites leaving the cell soma as 0–22°, 22.5–67°, or 67.5–90°, where 90° was perpendicular to the GCL.

Images of 12 regions-of-interest (ROI; each 221.4 µm^2^) were taken per animal using a confocal laser scanning microscope (Leica), with Kr-Ar 488 and 568 nm excitation filters, 63× water-immersion objective, and 16× digital zoom. Images were taken on the proximal and distal part of apical dendrites of GFP+ cells in the inner and outer ML (iML and oML), respectively (Figure 1C) for analyses of dendritic spines, scaffolding proteins, adhesion molecules, pre-synaptic proteins, GABA, and post-synaptic glycine receptor. The iML and oML were defined as the first one-third (inner) and last one-third (outer) of the ML, respectively. To ensure the lack of bias, dendrites of all diameters were selected using the GFP channel only, by an observer blind to the treatment conditions. For each ROI, confocal images were analysed in ImageJ software [36], the brightness and contrast corrected and the noise reduced using the built-in ImageJ functions. Spines were defined as protrusions from the dendritic shaft and classified as filopodia (protrusion with long neck and no head), thin (protrusion with a neck and head <0.6 µm in diameter), stubby (protrusion with no obvious neck or head), or mushroom spines (protrusion with a neck and a head with a diameter >0.6 µm) [33]. Dendritic spine analysis included spine density (number of spines per micrometer) and classification of spine morphology. Clusters were defined as spherical Cy3+ areas still present after correction for background and with a minimum cluster size of 0.025 µm^2^. The cluster density (number of clusters per micrometer), cluster size (cluster area with a range of 0.025–0.912 µm^2^) and location of clusters (on dendritic spine or dendritic shaft, Figure 1D) of the synaptic proteins were analysed for each ROI. Co-localisation of clusters on the GFP+ dendrites was confirmed by orthogonal projections (Figure 1D). When counting the presynaptic synaptophysin and CCK terminals and GABA clusters on the GFP+ dendrites, only those that perfectly co-localized with either GFP+ dendritic spines or shafts were counted. Pre-synaptic terminals in close proximity to but not overlapping with the GFP+ dendritic tree were excluded. For analysis of NL-2, GABA, and gephyrin expression on mature cells, the total number of Cy3+ NL-2, GABA, and gephyrin clusters were counted for each ROI stack and expressed as clusters/µm^3^ on GFP-negative cells, after the subtraction of clusters co-localising with GFP. Because there are also adult-born neurons not transduced by the retroviral vector carrying GFP possibly in close vicinity to the GFP+ neurons, subtraction of clusters on GFP+ cells will not give the true value on mature neurons, but an estimation.

### Statistical analysis

Comparisons were performed using two-way analysis of variance (ANOVA), unpaired Student's t-test, Pearson's Correlation analysis or chi-squared test. Data are presented as means ±SEM, and differences considered significant at p<0.05.

## Results

### Neurons formed in response to partial status epilepticus exhibit only subtle changes in distribution and morphology

**Figure 2:**
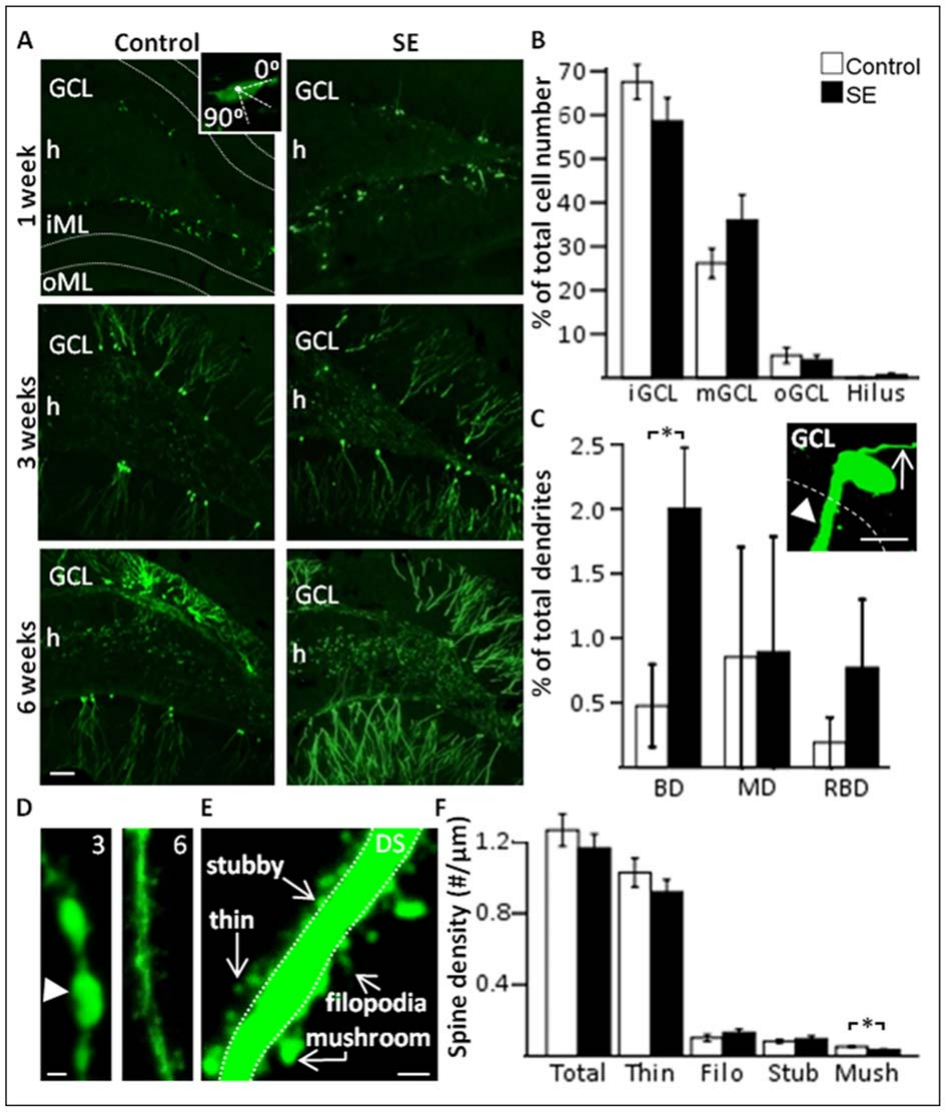
Neurons born in a partial SE (pSE)-induced environment develop with modest changes in gross morphology and dendritic spines. *A*, Morphological and temporal development of GFP+ new cells formed after partial SE or in controls. Inset: horizontal polarisation compared to granule cell layer (GCL) of a GFP+ cell body at one week. *B*, Percentage of GFP+ cell bodies in inner, middle, and outer GCL (iGCL, mGCL, and oGCL, respectively), and the hilus at six weeks. *C*, Percentage of basal (BD), medial (MD) or recurrent basal dendrites (RBD) on GFP+ cells at six weeks, showing an increase in BD in pSE animals. Inset: GFP+ cell showing a BD projecting towards the hilus (arrow head) and an apical axon directed towards the ML (arrow). *D*, Photomicrograph showing representative dendritic beading at three weeks (arrow head) but not at six weeks. *E*, Representative image showing examples of the different types of dendritic spines in relation to the dendritic shaft (DS). *F*, Spine density on GFP+ dendrites from control and pSE animals, showing a decrease in number of mushroom spines. Means ± SEM, n = 7 for each group (morphological analysis), n = 8 control and n = 9 pSE group (dendritic spine analysis). *, P<0.05 unpaired t-test compared to control group. Scale bars are 50 µm (*A*), 10 µm (*C*), and 1 µm (*D*, *E*).

At one week after the new GFP+ cells had been formed, their cell bodies were primarily located in the inner GCL and SGZ in both pSE and non-stimulated control animals (control 92±4 vs SE 80±4%). It has been reported that the polarity of the cell body changes from 0° to 90° relative to the granule cell layer during granule cell maturation in healthy animals [33]. Here, we found that after pSE, fewer GFP+ cell bodies were oriented at 0° (control 67±6 vs pSE 43±5%) and more at 45° in relation to the GCL (control 19±3 vs pSE 34±5%) (Figure 2A), suggesting subtle alterations in gross morphology. In accordance with previous findings in normal animals [37], the dendritic tree extended into the inner but not yet the oML in both groups (Figure 2A). No differences were observed between pSE and control animals when estimating the number of apical, basal, medial (originating from the medial side of the cell body) or recurrent basal dendrites, or axons originating from the basal, medial or apical sides of the cell soma (data not shown). Aberrantly located hilar GFP+ cells were not observed in non-stimulated control animals, but in 4 of 7 pSE animals (a total of 10 cells).

Three weeks after cell formation, the majority of GFP+ cell bodies were now located in the inner and middle GCL in both groups (inner GCL: control 64.1±7.6 vs pSE 40.8±11.4; middle GCL: control 32.1±6.8 vs SE 57.0±12.4%), and oriented at 90° angle in relation to the GCL, without differences between the groups (control 56.1±7 vs pSE 60.7±18%). The dendritic tree extended into the oML (Figure 2A) and the total number of dendrites per cell body (control 1.1±0.06 vs pSE 1.2±0.19), and percentage of apical (control 94.5±2.6 vs pSE 99.2±0.8), basal (control 4.2±2.8 vs pSE 0.6±0.6), medial (control 0 vs pSE 0.2±0.2), and recurrent basal dendrites (control 1.3±1.3 vs pSE 0) were similar in the two groups. Axons originated primarily from the basal (control 82.4±6.0 vs pSE 80.7±10.7%) and medial soma (control 17.6±6.0 vs pSE 18.5±10.9%), and rarely from the apical side (control 0 vs pSE 0.8±0.8%). Aberrantly located hilar GFP+ cells were not observed in control animals but were present in two out of seven pSE animals (a total of 4 cells).

Six weeks after the cells had been born, the majority of the new cell bodies were still located in the inner and middle GCL (Figure 2B) and oriented at a 90° angle in relation to the GCL in both groups (control 66.4±5.1 vs pSE 54.9±3.3%). However, the dendritic tree in both groups exhibited more extensive arborization compared to cells analyzed at the three week time point (Figure 2A). No differences were detected in the total number of dendrites per cell body (control 1.3±0.03 vs pSE 1.3±0.07) or the percentage of apical (control 98.3±1.3 vs pSE 96.3±1.0), medial or recurrent basal dendrites (Figure 2C), but the percentage of hilar basal dendrites was higher in pSE animals compared to controls (Figure 2C), consistent with previous studies [5], [38]. Dendritic beading, which may reflect an immature dendritic morphology [10], was frequently observed in cells at three weeks of age, but rarely at the six week time point. Beading at three weeks was not affected by pSE (beaded dendrites: control 62.5 vs pSE 60%) (Figure 2D), which is in contrast to what has previously been suggested [39]. Axons analyzed in both groups originated primarily from the basal (control 79.1±7.8 vs pSE 87.8±3.4%) or medial soma (control 15.6±6.5 vs pSE 11.8±3.2%), and rarely from the apical side (control 5.3±3.7 vs pSE 0.4±0.4%) (Figure 2C, inset). Four aberrantly located GFP+ cells were observed in the hilus in two out of seven pSE animals while none were detected in controls.

**Figure 3:**
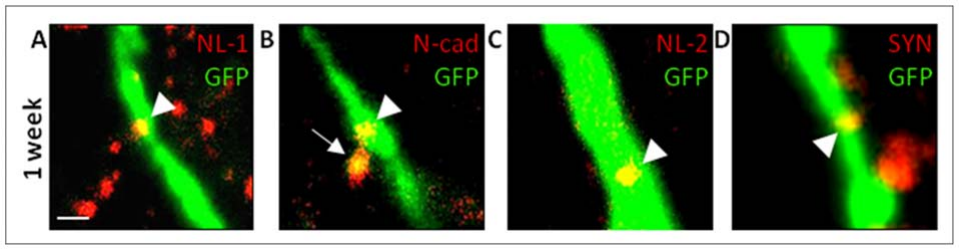
Expression of adhesion molecules in the ML on GFP+ dendrites of one-week-old cells formed after pSE or in control group. Representative photomicrographs of NL-1 (*A*), NL-2 (*C*), and synaptophysin (*D*) clusters on dendritic shafts (arrow heads). *B*, N-cadherin clusters on dendritic spine (arrow) and shaft (arrow head). Scale bar is 1 µm.

Dendritic spines are considered to be the main sites for excitatory input onto neurons, and can be divided into either immature phenotypes; filopodia and stubby spines, or mature phenotypes; thin and mushroom spines (Figure 2E). At one week, spines were very rare but occasionally seen on GFP+ dendrites in the iML in both pSE and control group (Figure 3B). This is slightly earlier than previously reported in vivo [33], even though the viral labeling procedure was the same in both studies. At three weeks, all spine types were present with no differences in spine density, neither when iML and oML were pooled (control 0.84±0.07 vs pSE 0.75±0.09 spines/µm), nor when they were analyzed separately (iML: control 0.89±0.10 vs pSE 0.80±0.11, oML: control 0.86±0.08 vs pSE 0.70±0.09 spines/µm). In addition, there were no differences in density of different spine types (filopodia: control 0.05±0.01 vs pSE 0.06±0.02, stubby: control 0.07±0.008 vs pSE 0.07±0.01, thin: control 0.59±0.06 vs pSE 0.68±0.06, mushroom: control 0.04±0.007 vs pSE 0.03±0.005 spines/µm). In both the pSE and control group, the density of spines on the GFP+ dendrites increased significantly between three and six weeks (control: 3 weeks 0.84±0.06 vs 6 weeks 1.48±0.12, pSE: 3 weeks 0.75±0.09 vs 6 weeks 1.24±0.10 spines/µm), but with no differences in spine density between the two groups, neither when the iML and oML were pooled, nor when they were analysed separately (iML: control 1.49±0.15 vs pSE 1.2±0.11, oML: control 1.50±0.14 vs pSE 1.36±0.10 spines/µm) on GFP+ dendrites at six weeks. Consistent with other studies [5], [32], [33] the density of mushroom spines was low compared to other spine types. However, we did detect a significant decrease specifically in mushroom spine density on the GFP+ cells formed after pSE at the six week time-point (iML and oML pooled: Figure 2F).

### Neurons formed in response to partial status epilepticus develop decreased scaffolding protein but normal adhesion molecule expression at excitatory synapses at six weeks of age

We quantified the cluster density and size of adhesion molecules and scaffolding proteins on GFP+ apical dendrites of new neurons in both the iML and oML. Mature granule cells primarily receive inhibitory afferents from interneurons and excitatory afferents from the contralateral hippocampus onto their dendrites in the iML, while in oML, afferents from the entorhinal cortex are the main excitatory input [40].

**Figure 4:**
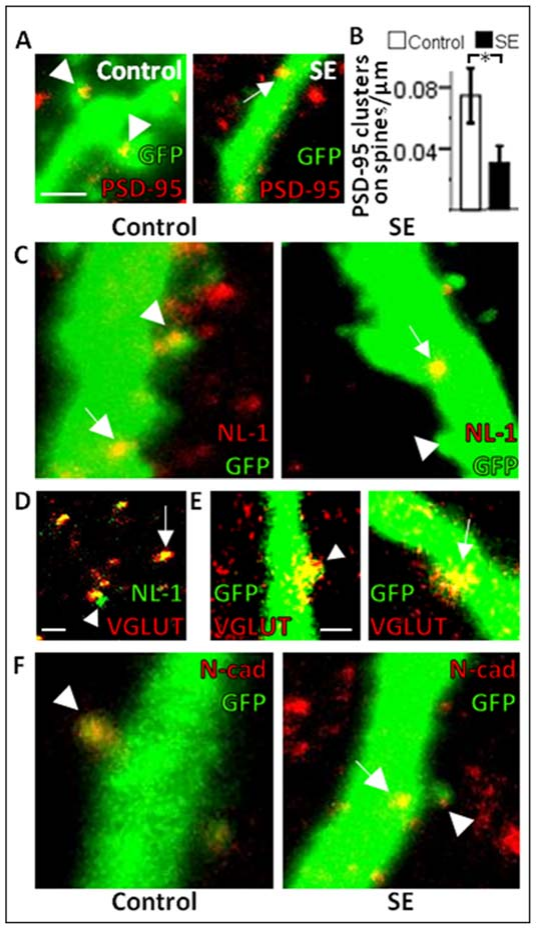
Six-week-old neurons formed after a pSE insult show decreased scaffolding protein expression but unaltered expression of adhesion molecules important at excitatory synapses. Representative photomicrographs of PSD-95 (*A*), NL-1 clusters (*C*), and N-cadherin (*F*), on GFP+ dendritic spines (arrow heads) and shafts (arrows). Note the distribution of NL-1 clusters mainly on the dendritic shaft. *B*, Cluster density of PSD-95 expression on dendritic spines (iML and oML pooled). *D*, Image showing co-localization of NL-1 clusters with VGLUT1 (arrow) in ML confirming the presence of NL-1 at excitatory synapses. A few clusters did not co-localize (arrow head). *E*, Images showing VGLUT1 located on a spine (arrow head) and on the dendritic shaft (arrow) of GFP+ dendrites. Means ± SEM, n = 5 control and n = 4 pSE group *, P<0.05 unpaired t-test compared to control group. Scale bars are 1 µm (in *A* for *A*, *C*, *F* and in *D* and in *E*).

**Figure 5:**
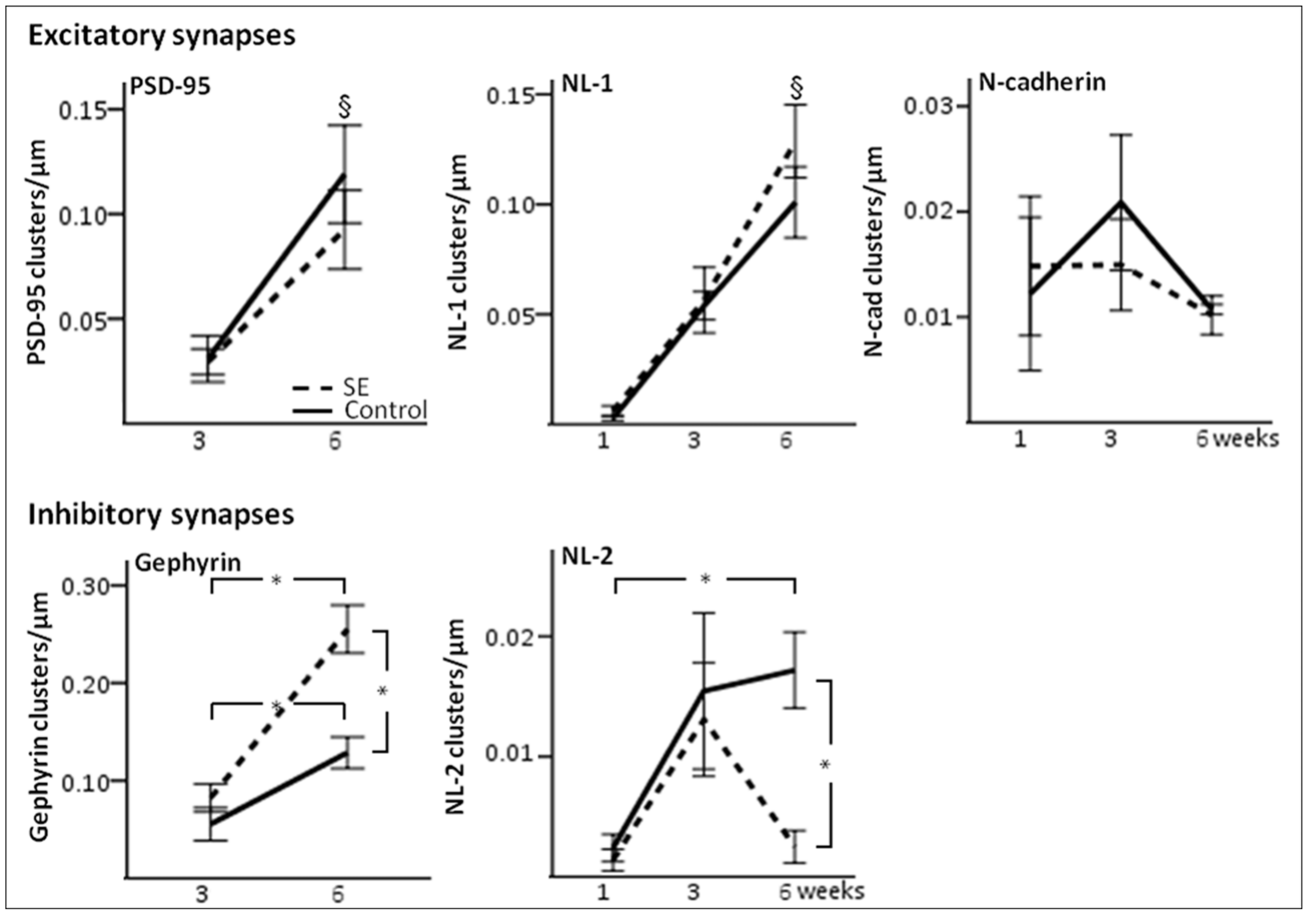
Temporal expression of scaffolding proteins and adhesion molecules at excitatory and inhibitory synapses in one, three, and six-week-old neurons formed in control or after pSE. At the excitatory synapses, both total PSD-95 and NL-1 cluster density increased with time. In contrast, there were no significant differences over time in total N-cadherin cluster density. Also at the inhibitory synapses, both total gephyrin and NL-2 cluster density increased continuously with time in the control group. However, at six weeks in the pSE group, the density of gephyrin clusters was higher compared to control group, whilst the NL-2 cluster density was reduced. §, P<0.05 two-way ANOVA comparing time-points in both pSE and control group. *, P<0.05 unpaired t-test comparing pSE to control group or between time-points.

We first determined the expression of the post-synaptic excitatory scaffolding protein PSD-95, which is concentrated at glutamatergic synapses [41]. We found PSD-95 clusters on both GFP+ dendritic spines and shafts at three and six weeks after labeling in both pSE and control animals (Table 1, Figure 4A), providing evidence that a substantial portion of the excitatory synapses are located on the dendritic shafts of the new cells in both groups. There were no significant differences between the pSE and control group in PSD-95 cluster density or size on dendritic spines and shafts of GFP+ new neurons, when analyzing iML and oML separately, three or six weeks after labeling (Table 1). Both groups exhibited a similar increase in total PSD-95 cluster density (dendritic spines and shaft and iML and oML pooled) with time (Figure 5). However, the number of PSD-95 clusters (iML and oML pooled) specifically located on spines, predominantly on thin spines, was reduced on GFP+ cells in the pSE group at six weeks (Figure 4B). The number of PSD-95 clusters on spines correlated significantly to the total number of spines, suggesting that a moderate loss of spines in the pSE group may explain the decrease in PSD-95 clusters at six weeks after labeling.

**Table 1:** Quantitative analysis of the expression of scaffolding proteins and adhesion molecules at excitatory synapses in controls and after partial status epilepticus.

	weeks	group	CD spine iML	CD spine oML	CD shaft iML	CD shaft oML	CS iML	CS oML
**PSD-95**	3	c	0.01±0.005	0.01±0.009	0.02±0.007	0.01±0.009	0.18±0.03	0.17±0.01
		*pSE*	*0.01±0.002*	*0.02±0.007*	*0.02±0.007*	*0.02±0.007*	*0.16±0.02*	*0.20±0.05*
	6	c	0.06±0.02	0.09±0.03	0.05±0.02	0.04±0.02	0.11±0.01	0.12±0.01
		*pSE*	*0.03±0.01*	*0.03±0.004*	*0.09±0.03*	*0.05±0.01*	*0.12±0.006*	*0.11±0.01*
**NL-1**	1	c	0	-	0.003±0.001	-	0.08±0.008	-
		*pSE*	*0.001±0.001*	*-*	*0.005±0.002*	*-*	*0.12±0.01*	*-*
	3	c	0.02±0.007	0.01±0.004	0.04±0.01	0.04±0.006	0.09±0.003	0.11±0.009
		*pSE*	*0.01±0.006*	*0.006±0.003*	*0.06±0.02*	*0.03±0.01*	*0.09±0.004*	*0.09±0.007*
	6	c	0.02±0.01	0.03±0.008	0.12±0.02	0.03±0.008	0.09±0.008	0.09±0.003
		*pSE*	*0.03±0.01*	*0.03±0.008*	*0.13±0.02*	*0.03±0.008*	*0.10±0.03*	*0.10±0.006*
**N-cadherin**	1	c	0.006±0.006	-	0.002±0.002	-	0.17±0.17	-
		*pSE*	*0.004±0.004*	*-*	*0.003±0.003*	*-*	*0.21±0.05*	*-*
	3	c	0.01±0.004	0.02±0.01	0.007±0.004	0.004±0.002	0.14±0.04	0.15±0.01
		*pSE*	*0.01±0.004*	*0.002±0.002*	*0.004±0.002*	*0.006±0.003*	*0.20±0.04*	*0.14±0.006*
	6	c	0.006±0.002	0.02±0.007	0	0.004±0.003	0.14±0.03	0.20±0.01
		*pSE*	*0.006±0.002*	*0.003±0.002*	*0.004±0.004*	*0.004±0.003*	*0.17±0.03*	*0.12±0.03*

Scaffolding protein (PSD-95) and adhesion molecule (NL-1 and N-cadherin) expression at excitatory synapses on GPF+ dendrites of one, three, and six-week-old adult-born neurons, formed after pSE and in controls (c). Cluster density (CD; clusters/µm) is given for iML and oML, on dendritic spines and shafts. Cluster size (CS; µm^2^) is given for iML and oML, dendritic shafts and spines pooled. Values are mean ± SEM.

During development, sub-cellular distribution of PSD-95 resembles that of NL-1, a post-synaptic adhesion molecule primarily located on excitatory synapses [42]. NL-1 is involved in the morphological and functional presynaptic differentiation of synapses [17], [43]. Previous studies have found the majority of NL-1 clusters on the dendritic spines of mature neurons [42], but we observed most NL-1 clusters on the GFP+ dendritic shafts of the newly formed neurons in both control and pSE animals (Table 1, Figure 4C, 5A) using two different antibodies (clusters on shafts: Ab#1: control 78±6% vs pSE 80±7%, Ab#2: control 74±2% vs pSE 81±5%). Also the NL-1 cluster density and size assessed separately in the iML at one, three, and six weeks and oML at three and six weeks, were similar in both groups (Table 1 for Ab#1). Furthermore, both the pSE and control group showed a continuous increase over time in total NL-1 cluster density (Figure 5). We confirmed that the NL-1 clusters were located at excitatory synapses by co-labeling with VGLUT1, a pre-synaptic vesicular marker of glutamatergic synapses. The qualitative description showed that the vast majority of VGLUT1+ terminals and NL-1 clusters co-localized in the ML (Figure 4D). Moreover, VGLUT1+ terminals were located on GFP+ dendritic spines at six weeks, as expected, but also on the dendritic shafts (Figure 4E).

We then characterized the expression of the adhesion molecule N-cadherin, which is found both pre- and postsynaptically. During development, N-cadherin is initially widely distributed but becomes localized primarily to excitatory synapses as the cell matures [44]. At one week after the new granule cells were labeled, N-cadherin clusters were occasionally observed and were then mainly present on spines of GFP+ dendrites in the iML (Figure 3B), with no differences in density or size between pSE and control animals (Table 1). Also at three and six weeks, no differences were seen between the two groups in the iML or the oML (Table 1). The majority of clusters were located on spines, especially thin spines (Figure 4F). No significant differences over time were detected when comparing total N-cadherin cluster density in pSE and control animals (Figure 5).

### Neurons formed in response to partial status epilepticus develop increased scaffolding protein but decreased adhesion molecule expression at inhibitory synapses at six weeks of age

**Figure 6:**
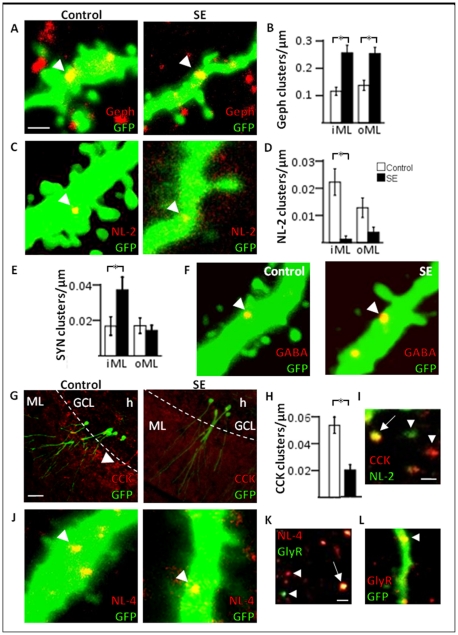
Six-week-old neurons formed after a pSE insult exhibit increased scaffolding protein expression, without an accompanying increase in GABAergic input or in the expression of adhesion molecules important at inhibitory synapses. Representative photomicrographs of gephyrin (*A*), NL-2 (*C*), and GABA (*F*) clusters on GFP+ dendritic shafts (arrow heads). Cluster density of gephyrin (*B*), NL-2 (*D*), synaptophysin (*E*) in the iML and oML. *G*, Representative images of CCK expression in GCL and ML. Note the increased CCK expression in the iML in the control image (arrow head). *H*, Cluster density of CCK in the iML. *I*, Photomicrograph of the co-localization of NL-2 and CCK clusters (arrow) in ML confirming the localization of NL-2 at inhibitory synapses. A few clusters did not co-localize (arrow heads). *J*, Representative images of NL-4 on GFP+ dendritic shafts (arrow heads). *K*, Image of NL-4 co-localizing with glycine receptors (arrow) showing the presence of NL-4 at glycinergic inhibitory synapses. Some clusters were not co-localized (arrow heads). *L*, Image showing the presence of glycine receptors on the dendritic shaft of newly formed GFP+ cells (arrow head). Means ± SEM, n = 8 control and n = 5 pSE group (gephyrin), n = 7 control and n = 8 pSE group (NL-2), n = 6 control and n = 8 pSE group (synaptophysin), n = 5 control and n = 4 pSE group (CCK); *, P<0.05 unpaired t-test compared to control group. Scale bars are 1 µm (in *A* for *A*, *C*, *F*, *J*), 20 µm (*G*) and 1 µm (*I* and in *K* for *K*, *L*).

Gephyrin is a post-synaptic scaffolding protein associated with clustering of GABA_A_ and glycine receptors at inhibitory synapses [45]. At three weeks after labeling, neither gephyrin cluster density nor size in iML and oML differed between the pSE and control group (Table 2) and both groups showed an increase in total gephyrin cluster density between three and six weeks (Figure 5). However at six weeks, pSE gave rise to an increase in density of gephyrin clusters compared to controls (Figure 5), which were also solely found on the dendritic shafts (Figure 6A) in both iML and oML (Table 2, Figure 6B). Gephyrin cluster size was unchanged (Table 2).

**Table 2:** Quantitative analysis of the expression of scaffolding proteins and adhesion molecules at inhibitory synapses in controls and after partial status epilepticus.

	weeks	group	CD shaft iML	CD shaft oML	CS iML	CS oML
**Gephyrin**	3	c	0.05±0.01	0.06±0.02	0.11±0.01	0.10±0.02
		*pSE*	*0.09±0.03*	*0.08±0.01*	*0.13±0.02*	*0.09±0.007*
	6	c	* **See ** **Fig 6**	* **See ** **Fig 6**	0.24±0.02	0.19±0.01
		*pSE*	* ***See *** ***Fig 6***	* ***See *** ***Fig 6***	*0.23±0.03*	*0.20±0.02*
**NL-2**	1	c	0.002±0.002	-	0.17±0.17	-
		*pSE*	*0.003±0.003*	*-*	*0.21±0.05*	*-*
	3	c	0.02±0.01	0.01±0.006	0.14±0.02	0.13±0.01
		*pSE*	*0.02±0.01*	*0.01±0.002*	*0.013±0.02*	*0.16±0.04*
	6	c	* **See ** **Fig 6**	0.01±0.004	0.14±0.02	0.10±0.01
		*pSE*	* ***See *** ***Fig 6***	*0.004±0.002*	*0.11±0.11*	*0.11±0.03*
**NL-4**	6	c	0.02±0.01	0.03±0.01	0.07±0.01	0.08±0.10
		*pSE*	*0.05±0.02*	*0.04±0.01*	*0.07±0.02*	*0.08±0.01*

Scaffolding protein (gephyrin) and adhesion molecule (NL-2 and NL-4) expression at inhibitory synapses, on GPF+ dendrites of one, three, and six-week-old adult-born neurons, formed after pSE and in controls (c). Cluster density (CD; clusters/µm) and cluster size (CS; µm^2^) are given for iML and oML, on dendritic shafts. Values are mean ± SEM.

* , P<0.05 unpaired t-test compared to control group (see Fig. 6).

The primary adhesion molecule at inhibitory synapses is NL-2 [46], which is thought to be involved in the morphological and functional presynaptic differentiation of synapses [17], [43] as well as in binding gephyrin at the postsynaptic densities [47]. Inhibitory synapses are located primarily on the dendritic shafts and not on spines [48]. In accordance, we found NL-2 clusters only on the dendritic shafts of the new cells in both control and pSE animals at one, three, and six weeks (Figure 3C, 6C). At one and three weeks, there were no differences in NL-2 cluster size or density in iML or oML between pSE and control animals (Table 2). However at six weeks, new neurons formed after pSE exhibited a significant reduction in NL-2 cluster density in the iML compared to controls (Table 2, Figure 6D), and the increase in total NL-2 cluster density with time, seen in the controls, did not occur in the pSE animals (Figure 5).

The decrease in NL-2 clusters at six weeks could possibly reflect less inhibitory presynaptic input to the new neurons, leading to postsynaptic alterations [49]. Co-localization of the presynaptic vesicle marker, synaptophysin, and GFP has previously been shown to indicate the presence of presynaptic terminals onto the newly-formed GFP+ cells [50]. Clusters of synaptophysin were occasionally present on the GFP+ dendrites of the new cells as early as one week in both the pSE and control group (Figure 3D). Since the NL-2 expression was specifically reduced at six weeks, we quantified the total synaptic input to the new neurons at this time point, using synaptophysin cluster density and size measurements. The number of synaptophysin clusters was increased in iML of pSE animals, whereas no group differences were observed in oML (Figure 6E). The difference was due to increased total number of clusters onto the dendritic shafts (control 0.009±0.002 vs SE 0.018±0.003 cluster/µm) and not on the spines (control 0.008±0.002 vs SE 0.008±0.002 cluster/µm). Synaptophysin is expressed on both inhibitory and excitatory presynaptic terminals apposed to the dendritic shafts, and a selective reduction in inhibitory presynaptic input is still possible. We therefore assessed the number of GABA clusters on the new neurons at six weeks (Figure 6F). However, there were no differences between groups in either cluster density or size in iML (cluster density: control 0.03±0.01 vs SE 0.02±0.01 cluster/µm, cluster size: control 0.11±0.02 vs SE 0.11±0.01 µm^2^) or oML (cluster density: control 0.01±0.01 vs SE 0.03±0.01 cluster/µm, cluster size: control 0.09±0.01 vs SE 0.14±0.03 µm^2^). Due to the discrepancy between the number of NL-2 and GABA clusters, we quantified a specific inhibitory input to the iML by counting the number of CCK-expressing interneuron terminals on the new neurons at six weeks. CCK-expressing processes were common throughout the iML in controls and appeared to be fewer in pSE animals (Figure 6G). All CCK clusters were found on dendritic shafts, suggesting the close apposition of CCK+ pre-synaptic terminals with inhibitory postsynaptic densities, and none on dendritic spines of GFP+ new neurons. Both cluster density (Figure 6H) and size (control 0.18±0.01 vs SE 0.13±0.01 µm^2^) were decreased on the GFP+ dendrites in the iML of pSE animals. CCK+ presynaptic terminals activate postsynaptic GABAergic receptors [51] and NL-2 is essential for GABA_A_ receptor clustering [52]. Virtually all CCK and NL-2 clusters co-localized (Figure 6I), supporting that NL-2 was confined to inhibitory synapses. Our finding implies a selective pSE-induced vulnerability of the CCK/NL-2 expressing synapses on the newly formed neurons at 6 weeks of age.

We then analysed the expression of NL-4, an adhesion molecule also present at inhibitory synapses, but in contrast to NL-2, primarily at glycinergic synapses [53]. We found NL-4 clusters on the dendritic shafts and none on dendritic spines of the GFP+ cells at six weeks (Figure 6J). However, no differences were found between the pSE and control group in NL-4 cluster density or size in the iML or oML (Table 2). The NL-4 clusters were co-localized with clusters of a postsynaptic glycine receptor (Figure 6K), and the vast majority of glycine receptor clusters were located on the dendritic shafts of the new neurons in both groups (Figure 6L).

### Neurons formed in response to a less-severe seizure insult do not exhibit changes in NL-2 and gephyrin expression at six weeks of age

In order to compare the synaptic changes on newly formed cells formed after pSE with those born after less severe seizures, we studied GFP+ cells born following a mild seizure insult associated with only a minor inflammatory response [32]. As previously described, neither gephyrin cluster density nor size were different on six-week-old newborn neurons formed after this milder seizure insult compared to control conditions [32]. Accordingly, we found that also NL-2 expression was unchanged in the newly formed cells, both when assessing cluster density (iML: control 0.01±0.01 vs SE 0.02±0.01 cluster/µm, oML: 0.02±0.01 vs SE 0.004±0.004 cluster/µm) and cluster size (iML: control 0.09±0.02 vs SE 0.12±0.03 µm^2^, oML: 0.11±0.01 vs SE 0.07±0.0 µm^2^).

### Newly formed and mature neurons exhibit similar changes in NL-2 and GABA but different gephyrin expression seven weeks after partial status epilepticus

**Figure 7:**
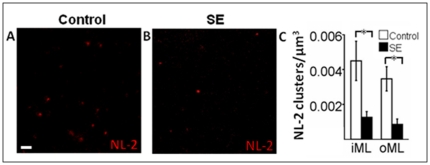
NL-2 expression on presumably mature GFP-negative cells seven weeks following pSE. *A*, Representative photomicrographs of NL-2 clusters in the ML. *B*, Cluster density of NL-2 expression in iML and oML. Means ± SEM, n = 7 control and n = 8 pSE group. Scale bar is 1 µm.

The density of synaptic protein clusters on GFP-negative cells was assessed to determine if the observed changes were specific for newly formed neurons. At seven weeks post pSE, similar to the six-week-old newborn neurons, there was a decrease in NL-2 cluster density in the iML, but the mature cells also showed a decrease in the oML (Figure 7). Moreover, GABA cluster density did not differ between the pSE and control group (iML: control 0.02±0.01 vs SE 0.03±0.01 cluster/µm^3^; oML: 0.02±0.01 vs SE 0.03±0.01 cluster/µm^3^), as for the newly formed cells. There were also no differences in gephyrin cluster density when comparing the pSE and control group (iML: control 0.08±0.02 vs SE 0.04±0.003 cluster/µm^3^, oML: 0.12±0.03 vs SE 0.06±0.01 cluster/µm^3^), which contrasted to the increased gephyrin expression on the six-week-old newborn neurons.

### Newly formed and mature neurons exhibit no changes in NL-2 or gephyrin expression three months after partial status epilepticus

To determine if the observed changes in inhibitory synaptic proteins observed at six weeks persisted when the new cells had matured further, we assessed the gephyrin and NL-2 cluster densities on GFP+ cells at three months of age, that is 3 months and 1 week after pSE. At this time point, no differences were seen in the NL-2 or gephyrin cluster densities or size when comparing with a control group (Table 3). In addition, the gephyrin and NL-2 cluster density on GFP-negative cells, presumably mature neurons, was also not different (Table 3).

**Table 3:** Quantitative analysis of neuroligin-2 and gephyrin expression at inhibitory synapses in three-month-old adult-born neurons and mature neurons.

	weeks	group	CD iML	CD oML	CS iML	CS oML
**NL-2 (GFP+ cells)**	12	c	0.01±0.01	0.002±0.002	0.11±0.01	0.11±0
		*pSE*	*0.02±0.002*	*0.002±0.002*	*0.14±0.04*	*0.09±0.004*
**NL-2 (GFP− cells)**	12	c	0.01±0.002	0.002±0.001	-	-
		*pSE*	*0.01±0.001*	*0.004±0.001*	*-*	*-*
**Gephyrin (GFP+ cells)**	12	c	0.03±0.02	0.04±0.03	0.12±0.01	0.13±0.01
		*pSE*	*0.04±0.02*	*0.05±0.02*	*0.13±0.01*	*0.12±0.01*
**Gephyrin (GFP− cells)**	12	c	0.02±0.01	0.02±0.01	-	-
		*pSE*	*0.02±0.003*	*0.03±0.01*	*-*	*-*

Expression of the adhesion molecule, NL-2, and the scaffolding protein, gephyrin, in three-month-old adult-born GFP+ cells and presumably mature GFP-negative cells. For GFP+ cells, cluster density (CD; clusters/µm) and cluster size (CS; µm^2^) are given for iML and oML, on dendritic shafts. For GFP− cells, cluster density (CD; clusters/µm^3^) is given for iML and oML in a 3D confocal stack of 0.2 µm-thick visual planes. Values are mean ± SEM. *, P<0.05 unpaired t-test compared to control group.

## Discussion

Adhesion molecules as well as other synaptic proteins regulate both synaptic establishments and spine shape, and synaptic transmission and strength [18], [20], [54]. Here we show that new hippocampal neurons formed after pSE develop altered expression of adhesion molecules and scaffolding proteins between 3 and 6 weeks of age at particularly inhibitory but also excitatory synapses. We report decreased postsynaptic clustering of PSD-95, specifically on dendritic spines, which may indicate a pSE-induced decreased excitatory connectivity. However, NL-1 and N-cadherin expression were not affected. Conversely, we found increased postsynaptic clustering of gephyrin, without an accompanying change in overall GABAergic input or NL-4/glycine receptor expression and with even a reduced NL-2/CCK expression, which could imply an increased but altered inhibitory connectivity. The differences in inhibitory sub-cellular protein expression were transient and had disappeared at three months of age. They also correlated with the magnitude or severity of the pathological conditions because following a less severe seizure insult with minor inflammation, no differences were observed. The change in gephyrin expression was specific to the newly formed cells, whereas the alterations in NL-2 expression were also evident on mature neurons.

Fewer mushroom spines and less PSD-95-expressing spines on new neurons formed after pSE are in contrast to previous reports describing a selective increase in mushroom spines and the occurrence of excitatory mossy fiber terminals apposed to spines after generalized SE [12], [13]. In addition, after kindling, a model of epileptogenesis, more stubby spines were detected on newly formed neurons [32]. However, in studies on presumably mature neurons after generalized SE, authors report down-regulation of PSD-95 expression in the adult hippocampus [55], an initial decrease in the total number of excitatory synapses in the iML, but a recovery to normal levels at later stages [56], [57]. A recent report describes even an increase in presumably excitatory synapses on the dendritic shafts of mature granule cells after generalized SE [56]. We could not find supporting evidence for such a compensatory increase in either shaft PSD-95, NL-1, or N-cadherin clusters on new neurons up to the age of 6 weeks. This diversity in spine numbers and excitatory synapses between different studies most likely depends on the age of the evaluated neurons, whether they are newly formed or mature, the severity of the pathology, and the sprouting capability of the surrounding network. The functional consequences of fewer mushroom spines and PSD-95 clusters reported here may suggest reduced excitation, which has previously been reported for these new neurons formed after pSE [5].

The reductions in spine number and PSD-95 expression on spines were not accompanied by reduced NL-1 or N-cadherin expression. Thus, because only about 20% of the NL-1 clusters were located on spines, NL-1 is probably less important for excitatory transmission within spines. Moreover, even if N-cadherin expression was mainly found on spines on the six-week-old neurons, its most essential role might be at earlier time-points when synapse assembly occurs. This idea is supported by previous reports describing the importance of N-cadherin in target recognition and synapse stabilization [25], [26], [27], In the present study, N-cadherin expression did also not increase between three and six weeks even if there was a substantial increase in spine density in both pSE and controls, with no significant differences between the groups. Due to high variation among animals, a minor decrease in the three- week-old neurons formed after pSE cannot be excluded (Figure 5).

In contrast to the reduced PSD-95 expression, gephyrin expression was increased, which is in agreement with increased inhibitory transmission previously reported for these new neurons [5]. The correlation between gephyrin cluster density or size and synaptic inhibitory transmission is further supported by two recent studies on new neurons formed either in an inflammatory environment without seizures [35], or in a mild seizure-induced environment with minor inflammation [32]. In the first study, both gephyrin cluster size and inhibitory transmission were increased, while in the second one, there was no change in gephyrin expression and only a minor decrease in inhibitory transmission. Moreover, we show here that also NL-2 expression was not altered in the less severe seizure model, further supporting a relationship between alterations in sub-cellular synaptic protein expression and inhibitory synaptic transmission in newly formed neurons.

Surprisingly, while the density of gephyrin clusters was increased, the GABA cluster density did not differ between the pSE and the control group, and we found a region-specific reduction in the number of NL-2 clusters on the dendritic segments within the iML. The NL-2 clusters co-localized with CCK-expressing terminals, which were also decreased in numbers, a reduction supported by previous reports showing over all fewer dentate hilar CCK-expressing interneurons after generalized SE in rats [58], [59]. However, also a general loss of inhibitory GABAergic interneurons has been found in the hippocampus of patients with temporal lobe epilepsy and in rodent seizure models [60]). In addition, the number of electron microscopically-identified GABAergic synapses per presumably mature granule cell and the number of gephyrin-immunoreactive puncta per dentate gurus are reduced five days following generalized SE in rats. This initial decrease was, though, converted into an increase at 12 weeks after SE, suggesting synaptogenesis of remaining GABAergic neurons [56]. Whether there is a seizure-induced loss or gain of GABAergic synapses may, therefore, be dependent on the capability and length of an adaptation period following an insult. In agreement, both the increase in gephyrin expression and the decrease in NL-2 expression in newly formed neurons were normalized at three month of age. Whether these transient changes were dependent on a delayed compensatory sprouting of remaining inhibitory terminals remains to be elucidated.

The transient increase in postsynaptic clustering of gephyrin without an accompanying change in overall GABAergic input and the reduction of NL-2 expression may reflect immature inhibitory transmission as the expression of these molecules returns to control levels after three months. In support of this assumption, during the development of GABAergic synapses on hippocampal neurons *in vitro*, postsynaptic clustering of gephyrin precedes the accumulation of NL-2 [61]. Furthermore, NL-2 deficient mice exhibit decreased numbers of inhibitory vesicular GABA transporter puncta but unaltered numbers of symmetric synapses in the hippocampus, suggesting that NL-2 deletion alone could impair the function of inhibitory synapses without decreasing their numbers [62]. Finally, NL-2 transfected cerebellar granule cells show mIPSCs with faster decay time, and NL-2 can accelerate GABAergic synapse maturation, through differential expression of postsynaptic GABA_A_ receptor subtypes [52]. We found here that the NL-2 cluster density was decreased, but whether also the previously observed action potential-dependent spontaneous IPSCs in new hippocampal neurons [5] have slower decay time when formed after pSE as compared to under physiological conditions is unclear. The simultaneous increase in numbers of synaptophysin-containing terminals onto the dendritic shafts, may further suggest the occurrence of immature inhibitory synapses on the new neurons, with pre-synaptic terminals apposed to gephyrin-containing postsynaptic densities, but yet without GABA release and NL-2-dependent adhesion. However, since neither NL-2 nor gephyrin expression remained increased at later stages of maturation, the increased synaptophysin expression on dendritic shafts is likely to reflect also changes in number of excitatory synapses on the newly formed neurons.

In contrast to NL-2, NL-4 expression did not change in the six-week-old neurons born after pSE. We found a clear co-localization of NL-4 with glycine receptor clusters on the new neurons in both groups, which implies that glycinergic inhibition is likely to exist in the new neurons under both pathological conditions and in the normal brain. Glycine receptor expression (cluster density and size in iML and oML) was not changed in the six-week-old neurons formed after pSE compared to control animals (unpublished observation), which supports the present NL-4 finding.

An increase in gephyrin expression and action potential-dependent spontaneous IPSCs, without increased presynaptic input may indicate ongoing homeostatic scaling of the inhibitory synapses. The theory of synaptic scaling suggests tuning of a response in order to counteract any change in excitability. Since the new neurons born following pSE encounter a hyperexcitable environment, a counteracting tuning up of inhibitory strength, specific for newborn neurons, is plausible. In comparison, cultured hippocampal neurons exposed to prolonged high-frequency depolarizing conditions develop increased GABAergic synaptic strength. The increased synaptic strength correlated in time with the presence of both post-synaptic GABAA-receptor and pre-synaptic GAD-65 expression [63]. Interestingly, the post-synaptic changes preceded the presynaptic [63], which could suggest the occurrence of ongoing inhibitory synaptic scaling in the new neurons at six weeks of age, as a reaction to the seizure-induced pathology.

Our results provide the first evidence that pathology-induced alterations in afferent synaptic input and functional connectivity of adult-born hippocampal neurons are associated with sub-cellular changes in the expression of synaptic adhesion molecules and scaffolding proteins. We have previously shown that new hippocampal granule cells formed after pSE receive less excitatory and more inhibitory synaptic drive onto their dendritic tree compared to physiological conditions [5]. Here we found less dendritic spines and PSD-95 clusters on spines without a compensatory increase of PSD-95 on dendritic shafts, which may imply reduced excitation. Conversely, we observed more gephyrin but fewer NL-2 clusters with no differences in NL-4 clusters and GABAergic input. This could reflect increased but immature inhibitory activity or perhaps homeostatic scaling of inhibitory synapses.

In future studies, assessment of adhesive and postsynaptic properties of inhibitory and excitatory synapses will be important for understanding how new neurons integrate in existing neural circuitries, and for revealing more specifically vulnerable synaptic contacts in different brain pathologies. Studies in humans and animal models have demonstrated structural abnormalities in dendrites, which could contribute to neuronal dysfunction, epileptogenesis and cognitive/neurological deficits in epilepsy [64]. Stabilizing the properties of synapses by targeting for instance adhesion molecules may become possible novel therapeutic approaches to reduce network hyperexcitability in epilepsy.

## References

[1] Eriksson PS, Perfilieva E, Bjork-Eriksson T, Alborn AM, Nordborg C (1998). Neurogenesis in the adult human hippocampus.. Nat Med.

[2] Dupret D, Revest JM, Koehl M, Ichas F, De Giorgi F (2008). Spatial relational memory requires hippocampal adult neurogenesis.. PLoS One.

[3] Sahay A, Scobie KN, Hill AS, O'Carroll CM, Kheirbek MA (2011). Increasing adult hippocampal neurogenesis is sufficient to improve pattern separation.. Nature.

[4] Bengzon J, Kokaia Z, Elmer E, Nanobashvili A, Kokaia M (1997). Apoptosis and proliferation of dentate gyrus neurons after single and intermittent limbic seizures.. Proc Natl Acad Sci U S A.

[5] Jakubs K, Nanobashvili A, Bonde S, Ekdahl CT, Kokaia Z (2006). Environment matters: synaptic properties of neurons born in the epileptic adult brain develop to reduce excitability.. Neuron.

[6] Parent JM, Yu TW, Leibowitz RT, Geschwind DH, Sloviter RS (1997). Dentate granule cell neurogenesis is increased by seizures and contributes to aberrant network reorganization in the adult rat hippocampus.. J Neurosci.

[7] Liu YW, Curtis MA, Gibbons HM, Mee EW, Bergin PS (2008). Doublecortin expression in the normal and epileptic adult human brain.. Eur J Neurosci.

[8] Laplagne DA, Esposito MS, Piatti VC, Morgenstern NA, Zhao C (2006). Functional convergence of neurons generated in the developing and adult hippocampus.. PLoS Biol.

[9] Ge S, Sailor KA, Ming GL, Song H (2008). Synaptic integration and plasticity of new neurons in the adult hippocampus.. J Physiol.

[10] Toni N, Teng EM, Bushong EA, Aimone JB, Zhao C (2007). Synapse formation on neurons born in the adult hippocampus.. Nat Neurosci.

[11] Parent JM, Lowenstein DH (2002). Seizure-induced neurogenesis: are more new neurons good for an adult brain?. Prog Brain Res.

[12] Murphy BL, Pun RY, Yin H, Faulkner CR, Loepke AW (2011). Heterogeneous integration of adult-generated granule cells into the epileptic brain.. J Neurosci.

[13] Jessberger S, Zhao C, Toni N, Clemenson GD, Li Y (2007). Seizure-associated, aberrant neurogenesis in adult rats characterized with retrovirus-mediated cell labeling.. J Neurosci.

[14] Overstreet-Wadiche LS, Bromberg DA, Bensen AL, Westbrook GL (2006). Seizures accelerate functional integration of adult-generated granule cells.. J Neurosci.

[15] Mohapel P, Ekdahl CT, Lindvall O (2004). Status epilepticus severity influences the long-term outcome of neurogenesis in the adult dentate gyrus.. Neurobiol Dis.

[16] Fritschy JM (2008). Epilepsy, E/I Balance and GABA(A) Receptor Plasticity.. Front Mol Neurosci.

[17] Chih B, Engelman H, Scheiffele P (2005). Control of excitatory and inhibitory synapse formation by neuroligins.. Science.

[18] Dalva MB, McClelland AC, Kayser MS (2007). Cell adhesion molecules: signalling functions at the synapse.. Nat Rev Neurosci.

[19] Washbourne P, Dityatev A, Scheiffele P, Biederer T, Weiner JA (2004). Cell adhesion molecules in synapse formation.. J Neurosci.

[20] Arikkath J, Reichardt LF (2008). Cadherins and catenins at synapses: roles in synaptogenesis and synaptic plasticity.. Trends Neurosci.

[21] Goda Y, Davis GW (2003). Mechanisms of synapse assembly and disassembly.. Neuron.

[22] Takeichi M, Abe K (2005). Synaptic contact dynamics controlled by cadherin and catenins.. Trends Cell Biol.

[23] Gascon E, Vutskits L, Kiss JZ (2010). The role of PSA-NCAM in adult neurogenesis.. Adv Exp Med Biol.

[24] Shan W, Yoshida M, Wu XR, Huntley GW, Colman DR (2002). Neural (N-) cadherin, a synaptic adhesion molecule, is induced in hippocampal mossy fiber axonal sprouts by seizure.. J Neurosci Res.

[25] Bozdagi O, Valcin M, Poskanzer K, Tanaka H, Benson DL (2004). Temporally distinct demands for classic cadherins in synapse formation and maturation.. Mol Cell Neurosci.

[26] Bamji SX, Shimazu K, Kimes N, Huelsken J, Birchmeier W (2003). Role of beta-catenin in synaptic vesicle localization and presynaptic assembly.. Neuron.

[27] Mendez P, De Roo M, Poglia L, Klauser P, Muller D (2010). N-cadherin mediates plasticity-induced long-term spine stabilization.. J Cell Biol.

[28] Fujita M, Aihara N, Yamamoto M, Ueki T, Asai K (2001). Regulation of rat hippocampal neural cadherin in the kainic acid induced seizures.. Neurosci Lett.

[29] Fasen K, Beck H, Elger CE, Lie AA (2002). Differential regulation of cadherins and catenins during axonal reorganization in the adult rat CNS.. J Neuropathol Exp Neurol.

[30] Lothman EW, Bertram EH, Bekenstein JW, Perlin JB (1989). Self-sustaining limbic status epilepticus induced by ‘continuous’ hippocampal stimulation: electrographic and behavioral characteristics.. Epilepsy Res.

[31] Racine RJ (1972). Modification of seizure activity by electrical stimulation. II. Motor seizure.. Electroencephalogr Clin Neurophysiol.

[32] Wood JC, Jackson JS, Jakubs K, Chapman KZ, Ekdahl CT (2011). Functional integration of new hippocampal neurons following insults to the adult brain is determined by characteristics of pathological environment.. Exp Neurol.

[33] Zhao C, Teng EM, Summers RG, Ming GL, Gage FH (2006). Distinct morphological stages of dentate granule neuron maturation in the adult mouse hippocampus.. J Neurosci.

[34] Schneider Gasser EM, Straub CJ, Panzanelli P, Weinmann O, Sassoe-Pognetto M (2006). Immunofluorescence in brain sections: simultaneous detection of presynaptic and postsynaptic proteins in identified neurons.. Nat Protoc.

[35] Jakubs K, Bonde S, Iosif RE, Ekdahl CT, Kokaia Z (2008). Inflammation regulates functional integration of neurons born in adult brain.. J Neurosci.

[36] Sheffield J (2007). ImageJ, A Useful Tool for Biological Image Processing and Analysis.. Microscopy and Microanalysis.

[37] Toni N, Sultan S (2011). Synapse formation on adult-born hippocampal neurons.. Eur J Neurosci.

[38] Spigelman I, Yan XX, Obenaus A, Lee EY, Wasterlain CG (1998). Dentate granule cells form novel basal dendrites in a rat model of temporal lobe epilepsy.. Neuroscience.

[39] Fiala JC, Spacek J, Harris KM (2002). Dendritic spine pathology: cause or consequence of neurological disorders?. Brain Res Brain Res Rev.

[40] Cotman CW, Matthews DA, Taylor D, Lynch G (1973). Synaptic rearrangement in the dentate gyrus: histochemical evidence of adjustments after lesions in immature and adult rats.. Proc Natl Acad Sci U S A.

[41] El-Husseini AE, Schnell E, Chetkovich DM, Nicoll RA, Bredt DS (2000). PSD-95 involvement in maturation of excitatory synapses.. Science.

[42] Song JY, Ichtchenko K, Sudhof TC, Brose N (1999). Neuroligin 1 is a postsynaptic cell-adhesion molecule of excitatory synapses.. Proc Natl Acad Sci U S A.

[43] Scheiffele P, Fan J, Choih J, Fetter R, Serafini T (2000). Neuroligin expressed in nonneuronal cells triggers presynaptic development in contacting axons.. Cell.

[44] Benson DL, Tanaka H (1998). N-cadherin redistribution during synaptogenesis in hippocampal neurons.. J Neurosci.

[45] Fritschy JM, Harvey RJ, Schwarz G (2008). Gephyrin: where do we stand, where do we go?. Trends Neurosci.

[46] Hoon M, Bauer G, Fritschy JM, Moser T, Falkenburger BH (2009). Neuroligin 2 controls the maturation of GABAergic synapses and information processing in the retina.. J Neurosci.

[47] Poulopoulos A, Aramuni G, Meyer G, Soykan T, Hoon M (2009). Neuroligin 2 drives postsynaptic assembly at perisomatic inhibitory synapses through gephyrin and collybistin.. Neuron.

[48] van Spronsen M, Hoogenraad CC (2010). Synapse pathology in psychiatric and neurologic disease.. Curr Neurol Neurosci Rep.

[49] Chubykin AA, Atasoy D, Etherton MR, Brose N, Kavalali ET (2007). Activity-dependent validation of excitatory versus inhibitory synapses by neuroligin-1 versus neuroligin-2.. Neuron.

[50] van Praag H, Schinder AF, Christie BR, Toni N, Palmer TD (2002). Functional neurogenesis in the adult hippocampus.. Nature.

[51] Ali AB, Thomson AM (2008). Synaptic alpha 5 subunit-containing GABAA receptors mediate IPSPs elicited by dendrite-preferring cells in rat neocortex.. Cereb Cortex.

[52] Fu Z, Vicini S (2009). Neuroligin-2 accelerates GABAergic synapse maturation in cerebellar granule cells.. Mol Cell Neurosci.

[53] Hoon M, Soykan T, Falkenburger B, Hammer M, Patrizi A (2011). Neuroligin-4 is localized to glycinergic postsynapses and regulates inhibition in the retina.. Proc Natl Acad Sci U S A.

[54] Sudhof TC (2008). Neuroligins and neurexins link synaptic function to cognitive disease.. Nature.

[55] Sun QJ, Duan RS, Wang AH, Shang W, Zhang T (2009). Alterations of NR2B and PSD-95 expression in hippocampus of kainic acid-exposed rats with behavioural deficits.. Behav Brain Res.

[56] Thind KK, Yamawaki R, Phanwar I, Zhang G, Wen X (2010). Initial loss but later excess of GABAergic synapses with dentate granule cells in a rat model of temporal lobe epilepsy.. J Comp Neurol.

[57] Boulland JL, Ferhat L, Tallak Solbu T, Ferrand N, Chaudhry FA (2007). Changes in vesicular transporters for gamma-aminobutyric acid and glutamate reveal vulnerability and reorganization of hippocampal neurons following pilocarpine-induced seizures.. J Comp Neurol.

[58] Arabadzisz D, Antal K, Parpan F, Emri Z, Fritschy JM (2005). Epileptogenesis and chronic seizures in a mouse model of temporal lobe epilepsy are associated with distinct EEG patterns and selective neurochemical alterations in the contralateral hippocampus.. Exp Neurol.

[59] Wyeth MS, Zhang N, Mody I, Houser CR (2010). Selective reduction of cholecystokinin-positive basket cell innervation in a model of temporal lobe epilepsy.. J Neurosci.

[60] Sun C, Mtchedlishvili Z, Bertram EH, Erisir A, Kapur J (2007). Selective loss of dentate hilar interneurons contributes to reduced synaptic inhibition of granule cells in an electrical stimulation-based animal model of temporal lobe epilepsy.. J Comp Neurol.

[61] Chiou TT, Bonhomme B, Jin H, Miralles CP, Xiao H (2011). Differential regulation of the postsynaptic clustering of gamma-aminobutyric acid type A (GABAA) receptors by collybistin isoforms.. J Biol Chem.

[62] Blundell J, Tabuchi K, Bolliger MF, Blaiss CA, Brose N (2009). Increased anxiety-like behavior in mice lacking the inhibitory synapse cell adhesion molecule neuroligin 2.. Genes Brain Behav.

[63] Rannals MD, Kapur J (2011). Homeostatic strengthening of inhibitory synapses is mediated by the accumulation of GABA(A) receptors.. J Neurosci.

[64] Wong M (2008). Stabilizing dendritic structure as a novel therapeutic approach for epilepsy.. Expert Rev Neurother.

